# Remodeling of extracellular matrix by normal and tumor-associated fibroblasts promotes cervical cancer progression

**DOI:** 10.1186/s12885-015-1272-3

**Published:** 2015-04-11

**Authors:** Alexandra Fullár, József Dudás, Lászlóné Oláh, Péter Hollósi, Zoltán Papp, Gábor Sobel, Katalin Karászi, Sándor Paku, Kornélia Baghy, Ilona Kovalszky

**Affiliations:** 1 1st Department of Pathology and Experimental Cancer Research, Semmelweis University, Budapest, Hungary; 2 Department of Otorhinolaryngology, Medical University Innsbruck, Innsbruck, Austria; 3 Tumor Progression Research Group, Hungarian Academy of Sciences, Budapest, Hungary; 4 Maternity Private Department Kútvölgyi Clinical Block, Semmelweis University, Budapest, Hungary; 5 2nd Department of Obstetrics and Gynecology, Semmelweis University, Budapest, Hungary; 6 CRUK/MRC Oxford Institute for Radiation Oncology, Department of Oncology, University of Oxford, Oxford, UK

**Keywords:** Cancer associated fibroblasts, Cervical cancer, Extracellular matrix remodeling, Integrins, Laminin-1, Stromal fibroblasts

## Abstract

**Background:**

Comparison of tissue microarray results of 29 cervical cancer and 27 normal cervix tissue samples using immunohistochemistry revealed considerable reorganization of the fibrillar stroma of these tumors.

Preliminary densitometry analysis of laminin-1, α-smooth muscle actin (SMA) and fibronectin immunostaining demonstrated 3.8-fold upregulation of laminin-1 and 5.2-fold increase of SMA in the interstitial stroma, indicating that these proteins and the activated fibroblasts play important role in the pathogenesis of cervical cancer. In the present work we investigated the role of normal and tumor-associated fibroblasts.

**Methods:**

*In vitro* models were used to throw light on the multifactorial process of tumor-stroma interaction, by means of studying the cooperation between tumor cells and fibroblasts. Fibroblasts from normal cervix and cervical cancers were grown either separately or in co-culture with CSCC7 cervical cancer cell line. Changes manifest in secreted glycoproteins, integrins and matrix metallo-proteases (MMPs) were explored.

**Results:**

While normal fibroblasts produced components of interstitial matrix and TGF-β1 that promoted cell proliferation, cancer-associated fibroblasts (CAFs) synthesized ample amounts of laminin-1. The following results support the significance of laminin-1 in the invasion of CSCC7 cells: 1.) Tumor-associated fibroblasts produced more laminin-1 and less components of fibrillar ECM than normal cells; 2.) The production of laminin chains was further increased when CSCC7 cells were grown in co-culture with fibroblasts; 3.) CSCC7 cells were capable of increasing their laminin production; 4.) Tumor cells predominantly expressed integrin α6β4 laminin receptors and migrated towards laminin. The integrin profile of both normal and tumor-associated fibroblasts was similar, expressing receptors for fibronectin, vitronectin and osteopontin. MMP-7 secreted by CSCC7 cells was upregulated by the presence of normal fibroblasts, whereas MMP-2 produced mainly by fibroblasts was activated in the presence of CSCC7 cells.

**Conclusions:**

Our results indicate that in addition to degradation of the basement membrane, invasion of cervical cancer is accomplished by the remodeling of the interstitial stroma, which process includes decrease and partial replacement of fibronectin and collagens by a laminin-rich matrix.

## Background

Tumor microenvironment has become the focus of intensive research as a potential target for cancer therapy [1]. In the normal epithelium, parenchymal cells and stromal components are physically separated by a basement membrane. The transition from normal epithelium to invasive carcinoma is preceded by, or is concomitant with, the activation of local host stroma [2]. Invasion occurs in close cooperation with stromal cells and the transformed epithelium [2,3]. Malignant progression impairs the integrity of the basement membrane resulting in the deterioration of its organized structure. Invasive tumor cells lose their epithelial characteristics and acquire metastatic phenotype. In this process a vast number of macromolecules are produced by stromal cells capable of influencing the microenvironment [4].

Fibroblasts are characteristic cell types in the microenvironment playing a prominent role in the pathology of solid tumors [5]. Cancer-associated fibroblasts (CAFs) within the reactive stroma express elevated amounts of extracellular matrix (ECM) proteins, proteases. These matrix metalloproteinases (MMPs) play an important role in the degradation of the basement membrane and stromal ECM initiating the invasion of malignant tumors [6,7]. During this process newly synthesized ECM proteins serve as a scaffold for motile tumor cells to move along, as well as providing structural support for angiogenesis [5].

To obtain invasive phenotype, cervical carcinoma cells may utilize stromal MMPs. MMP-7 and MMP-9 expressions can be induced in cancer cells, augmented by tumor-stromal cell interaction and possibly mediated by membrane-anchored and/or soluble factors [8]. Their expression has been shown to correlate with the amount of the hyaluronan (HA) receptor CD44 noted in low grade squamous cell carcinoma (SCC) [9,10].

Growth signals generated by the stromal cells of the tumor are mediated to the cancer cells by integrins. These cell surface receptors are indispensable for the cross-talk between cancer cells and tumorous stroma [4,11]. Integrin-mediated communication is pivotal in cell survival, proliferation, migration and tumor invasion [12].

In addition to thrombocytes, fibroblasts are the major sources of TGF-β1, a cytokine that is one of the most important regulators of ECM. TGF-β1 has a strategic role in the regulation of assembly and remodeling of extracellular matrix during cancer progression. Furthermore, the growth inhibitory action exerted by TGF-β1 on epithelial cells disappears after malignant transformation, which together with the process of EMT, changes the status of the growth factor from inhibitor to tumor promoter [13]. The TGF-β1 molecule is synthesized as an inactive multidomain complex and its activation occurs through multiple extracellular mechanisms that may involve proteases, thrombospondin-1 and integrins [14,15]. This implies that the interplay between tumor cells and fibroblasts can modulate the effect of growth factors that in turn exert their modified action on the tumor tissue [4,16].

In the current work we investigated the course of matrix remodeling in cervical SCC by studying the molecular components listed above. To this end, we completed an immunohistochemical analysis of paraffin-embedded cervical SCC sections and established co-culture models between normal or tumorous cervical fibroblasts and CSCC7 HPV-positive cervix SCC cells.

## Methods

### Tissues, cell lines and materials

A tissue microarray (TMA) was generated from 27 normal and 29 cancerous, formalin fixed and paraffin embedded tissue blocks taken from the vaginal portions of cervices removed by radical Wertheim hysterectomy (Table 1). All cases had previously been analyzed for HPV genotypes [17]. Tissue blocks were collected from the 1^st^ Department of Obstetrics and Gynecology of Semmelweis University, with permission and seal from the Semmelweis University Regional and Institutional Committee of Science and Research Ethics (TUKEB permit number: 95/1999). Representative normal and tumorous areas on hematoxylin- and eosin-stained (HE) sections (identified by an independent pathologist) were excised. Tissue cores corresponding to the marked areas were used to assemble a TMA block which was then sliced, counterstained with hematoxilin and immunostained.

**Table 1:** **Patient data to TMA**

Patient	Age	FIGO stage	Histology	HPV positivity
**1**	37	II/A	Adenosquamous carcinoma	-
**2**	51	I/B	Squamous cell carcinoma	-
**3**	42	II/A	Squamous cell carcinoma	-
**4**	48	II/B	Squamous cell carcinoma	HPV18
**5**	39	II/B	Squamous cell carcinoma	HPV 16, 18
**6**	41	II/A	Squamous cell carcinoma	HPV16
**7**	38	II/B	Squamous cell carcinoma	HPV16
**8**	59	II/B	Squamous cell carcinoma	HPV16
**9**	39	II/B	Squamous cell carcinoma	HPV16
**10**	39	I/B	Squamous cell carcinoma	HPV16
**11**	71	II/A	Clear cell carcinoma	HPV16
**12**	51	II/A	Squamous cell carcinoma	HPV16
**13**	44	II/B	Squamous cell carcinoma	HPV16
**14**	42	II/A	Squamous cell carcinoma	HPV16
**15**	45	II/B	Squamous cell carcinoma	HPV16
**16**	32	I/B	Squamous cell carcinoma	HPV16
**17**	55	II/B	Squamous cell carcinoma	-
**18**	56	II/B	Squamous cell carcinoma	HPV16
**19**	57	II/B	Squamous cell carcinoma	HPV16
**20**	44	II/B	Squamous cell carcinoma	HPV16
**21**	35	I/B	Squamous cell carcinoma	HPV16
**22**	66	II/B	Squamous cell carcinoma	HPV16
**23**	57	II/A	Squamous cell carcinoma	HPV16
**24**	38	II/B	Squamous cell carcinoma	HPV16
**25**	40	II/B	Squamous cell carcinoma	HPV16
**26**	52	II/B	Squamous cell carcinoma	HPV16
**27**	62	II/B	Squamous cell carcinoma	HPV16
**28**	57	II/B	Squamous cell carcinoma	HPV16
**29**	54	II/B	Squamous cell carcinoma	HPV16

Fresh surgical specimens obtained from radical Wertheim hysterectomy were sent for routine pathology service to the 1^st^ Department of Pathology and Experimental Cancer Research from the Maternity Private Department of the Kútvölgyi Klinikal Block of Semmelweis University. Fibroblasts from normal and tumorous regions of uterine cervix not used for diagnosis were obtained from explant cultures. The surgical material was collected and used based on approval by the Semmelweis University Regional and Institutional Committee of Science and Research Ethics (TUKEB permit number: 95/1999). CSCC7 HPV16 positive cervical cancer cells, derived from a case of planocellular cervical cancer, were the gift of G. Gorter from Leiden University [18]. These cells exhibit a clear epithelial morphology and form nests when grown in monoculture. They are positive for pan-cytokeratin but negative for vimentin. In contrast, fibroblasts are vimentin positive, pan-cytokeratin negative cells displaying spindle-like morphology, with elongated, oval nuclei.

Materials and consumables used in cell cultures and the general chemicals used for the experiments were purchased from Sigma-Aldrich Co. (St. Louis, MO, USA), SARSTEDT AG&Co (Nümbrecht, Germany) and Merck (Darmstadt, F. R. Germany).

### Tissue microarray, histochemistry, immunohistochemistry and immunocytochemistry

TMA slides were immunostained for α-smooth muscle actin (SMA), laminin-1, laminin-5 and fibronectin. Subsequently, they were scanned with a Scan Scope CS2 (Aperio Technologies Inc., Vista, CA, USA) and analyzed by MAN-0023 Color Deconvolution Algorithm Positive Pixel Count Analysis software (Aperio Technologies Inc.). Staining intensities were measured only in stromal areas, and percentage of positive and negative pixels were evaluated. Immunocyto- and immunohistochemistry procedures followed standard protocols [19]. Antibodies used are listed in Table 2. Cell nuclei were counterstained with TOTO-3 (Invitrogen by Life Technologies Co., Carlsbad, California, USA). Images were taken using MRC-1024 confocal laser scanning microscope (Bio-Rad Laboratories GmbH, Münich, Germany).

**Table 2:** **Antibodies used in the present study**

**Primary antibodies**	**Host**	Manufacturer*	**Cat. no.**	**Dilution IHC/ IF**	**Dilution WB/DB**
β-Actin	Rabbit polycolnal IgG	Cell Signaling	4967	-	1:1000
Laminin	Rabbit polycolnal IgG	Dako	Z0097	1:200	-
Laminin α1 (H-300)	Rabbit polycolnal IgG	Santa Cruz	sc-5582	-	1:200
Laminin β1 (H-300)	Rabbit polycolnal IgG	Santa Cruz	sc-5583	1:50	1:200
Laminin 5	Rabbit polycolnal IgG	Abcam	Ab14509	1:100	-
Laminin-5 (P3E4)	Mouse monoclonal IgG1	Santa Cruz	sc-13587	1:50	1:200
Fibronectin	Rabbit polycolnal IgG	Dako	A0245	1:100	-
Fibronectin (IST-9)	Mouse monoclonal IgG1	Santa Cruz	sc-59826	1:40	1:500
Smoth Muscle Actin colone 1A4	Mouse monoclonal IgG1	Dako	M0851	1:200	-
Thrombospondin 1 (A6.1)	Mouse monoclonal IgG1	Santa Cruz	sc-59887	-	1:250
Perlecan	Mouse monoclonal IgG1	Zymed	13-4400	-	1:500
Collagen I	Rabbit polycolnal IgG	Abcam	ab34710	-	1:1000
Collagen III	Rabbit polycolnal IgG	Abcam	Ab7778	-	1:1000
Anti-Human Collagen IV	Mouse monoclonal IgG1	DakoCytomation	M 0785	-	1:200
Anti-Human MMP-7	Mouse monoclonal IgG_1K_	Chemicon	MAB3315	-	1:500
TIMP1 (D10E6)	Rabbit monoclonal IgG	Cell Signaling	8946	-	1:1000
TIMP3 (D74B10)	Rabbit monoclonal IgG	Cell Signaling	5673	-	1:1000
Integrin α4	Rabbit polycolnal IgG	Cell Signaling	4600	-	1:1000
Integrin α5	Rabbit polycolnal IgG	Cell Signaling	4705	-	1:1000
Integrin α6	Rabbit polycolnal IgG	Cell Signaling	3750	-	1:750
Integrin αV	Rabbit polycolnal IgG	Cell Signaling	4711	-	1:1000
Integrin β1	Rabbit polycolnal IgG	Cell Signaling	4706	-	1:1000
Integrin β3	Rabbit polycolnal IgG	Cell Signaling	4702	-	1:100
Integrin β4	Rabbit polycolnal IgG	Cell Signaling	4707	-	1:1000
CD44	Mouse monoclonal IgG2a	Antibodies online	ABIN96695	1:100	1:500
CD151 (PETA-3)	Mouse monoclonal IgG2b	Novocastra™ Leica Biosystems	NCL-CD151	1:50	-
**Secondary antibodies**	**Host**	Manufacturer*	**Cat. no.**	**Dilution IHC/IF**	**Dilution WB/DB**
Anti-mouse Ig/HRP	Goat polyclonal	DakoCytomation	P0447	-	1:2000
Anti-rabbit Ig/HRP	Goat polyclonal	DakoCytomation	P0448	-	1:2000
Alexa Fluor® 647 anti-mouse IgG (H + L)	Donkey polyclonal	Invitrogen	A31571	1:200	-
Alexa Fluor® 488 anti-mouse IgG (H + L)	Donkey polyclonal	Invitrogen	A21202	1:200	-
Alexa Fluor® 647 anti-rabbit IgG (H + L)	Goat polyclonal	Invitrogen	A21244	1:200	-
Alexa Fluor® 568 anti-rabbit IgG (H + L)	Goat polyclonal	Invitrogene	A11011	1:200	-

*Cell Signaling Technology. Danvers. MA; DakoCytomation. Dako North America Inc. Carpinteria. CA. USA; Santa Cruz Biotechnology. Inc.. Santa Cruz. CA. USA; Zymed Laboratories Inc.. South San Francisco. CA. USA; Abcam plc. Cambridge. UK; Chemicon International Inc.. Temecula. CA. USA; Diagnostic BioSystems. Pleasanton. CA. USA; Antibodies-online Inc.. Atlanta. GA. USA; Leica Biosystems Newcastle Ltd. Newcastle. UK; Invitrogen by Life Technologies. Carlsbad. California. USA;

### Generation of cell cultures

Tumorous and normal areas of surgical specimens taken from the same patient and not used for diagnosis were excised and cut into small pieces and placed into six-well tissue culture dishes containing Cytogen Amnio Grow Plus medium (CytoGen GmbH, Sinn, Germany), optimized for development of primary cell culture. Fibroblasts were allowed to grow till the third passage and were then routinely transferred into DMEM-low glucose medium supplemented with 10% fetal bovine serum (FBS), 2 mM L-glutamine, 100 units/mL penicillin and 100 μg/mL streptomycin. Normal fibroblasts are hereinafter referred to as NF, fibroblasts derived from tumorous areas as TF. Purity of the fibroblast cultures was tested by means of vimentin and citokeratin immunostaining. On average, <3% of epithelial contamination was found in the established fibroblast cultures. CSCC7 cells were routinely cultured in RPMI-1640 medium supplemented with 10% FBS, 2 mM L-glutamine, 1 mM sodium pyruvate, 100 units/mL penicillin and 100 μg/mL streptomycin. All cell lines were cultured in a humidified 95% air/5% CO_2_ incubator at 37°C. The cell lines used were between the 8^th^ and 12^th^ passage, within which period both fibroblasts and CSCC7 cells proved to be stable.

### Co-culture systems

Two models were used to study the interaction between fibroblasts and tumor cells. Direct co-cultivation allowed physical contact between cells, whereas in indirect co-cultures cells were separated by a transwell insert with a 0.45 μm pore size, allowing only molecular communication.

NF, TF, and CSCC7 cells were seeded in culture dishes 10 cm in diameter alone or in direct co-cultures with 5×10^5^ cells/dish density in a 1:1 (v/v) mixture of DMEM-low glucose and RPMI-1640 supplemented with 5% FBS. Seventy two hours after seeding, the FBS content was reduced to 0.3% and cells were incubated for 24 h. Conditioned culture media (CCMs) and cell layers (CLs) were then harvested and frozen for further protein analyses. Samples from direct co-cultures are indicated as NF + CSCC7 and TF + CSCC7.

Indirect co-cultures were set up as follows: fibroblasts were seeded in 6-well plates (Corning Incorporated Life Sciences, Acton, MA, USA) at a density of 2.5×10^4^ cells/well in DMEM-low glucose with 10% FBS. CSCC7 cells were placed in Transwell® polyester membrane inserts (Corning) with 0.45 μm filter pore size at a density of 5×10^4^ cells/insert in RPMI-1640 with 10% FBS. Forty-eight hours after seeding CSCC7 containing inserts were placed in fibroblast containing wells followed by addition of 1:1 (v/v) mixture of DMEM-low glucose and RPMI-1640 supplemented with 5% FBS to the indirect co-culture and to control cells growing alone. Seventy-two hours later the FBS content was reduced to 0.3% for another 24-h incubation period. CCMs and CLs were then collected and frozen for subsequent dot blot, gelatin zymography and ELISA assays. This treatment of the cells minimized the potential disturbing effects of serum metalloproteinases [20]. *NF*/CSCC7 and *TF*/CSCC7 indicate fibroblast samples isolated from the bottom of the filter compartment, whereas NF/*CSCC7* and TF/*CSCC7* designate tumor cell samples isolated from the inserts of indirect co-culturing plates.

### Proliferation assay

The principles of sulforhodamine B (SRB) colorimetric assay were described earlier [21]. This protocol was used in the current study with the following modifications. Fibroblasts or CSCC7 cells were seeded in 96 well plates at densities of 2.5×10^3^ or 3.5×10^3^ cells/well in 200 μL complete growth medium. All experimental conditions were run in 8 or 16 parallel samples. After counting, viable cells were let to seed and attach. Zero time point was considered three hours later after all cells were attached. SRB measurements were carried out at the time points of 0, 24, 48, 72 and 96 h. Cells were originally grown in the presence of 5% FBS, but to observe the potential negative effects of serum starvation applied in the last 24 h of the co-culture experiments, the FBS concentration was decreased to 0.3% 24 h before harvesting the cells. To mimic the effects of co-cultivation on cell proliferation, fibroblasts were allowed to grow with CCM of tumor cells, and the latter with CCM of fibroblasts. Specifically, the culture medium contained 50% regular and 50% conditioned medium that was conditioned for 48 h and sterile filtered. The incubation mixture was replaced every 24 h. To control these assays, cells were grown in DMEM-low glucose and RPMI-1640 mixed in 1:1 (v/v) ratio and supplemented with 5% FBS.

### Chemotaxis assay

Chemotaxis assays were performed in Boyden chambers as previously described [21]. The following materials were used as chemoattractants in separate assays: tissue culture medium with 10% FBS, medium conditioned by the two types of fibroblasts (NF and TF), fibronectin (from human plasma, Sigma, 25 μg/mL), and laminin-1 (from Engelbreth-Holm-Swarm murine sarcoma basement membrane, Sigma) diluted in serum-free medium to 25 μg/mL. The cells were treated with 10 μg/mL mitomycin C (Sigma) for five minutes in order to inhibit proliferation [22]. Two days after mitomycin C treatment, 5×10^4^ CSCC7 cells were seeded into the upper chambers of a 48-well Micro Chemotaxis Chamber (Neuro Probe, Gaithersburg, MD, USA) with medium containing 10% FBS and migration was allowed for 24 h. Cell migration toward each chemoattractant was measured in triplicate samples. Migrated cells were stained with toluidine blue with 3 random fields per well. Accordingly, 9 random fields per each chemoattractant were counted.

### Protein expression and activity measurements

#### Western and dot blot

For Western blot, cells were grown as indicated above in the co-culture system. CCMs were collected and cells were extracted by lysis buffer containing 20 mM HEPES pH 7.8, 10 mM KCl, 0.1 mM EDTA, 1 mM dithiothreitol, 1% (v/v) Nonidet P40 and protease inhibitory cocktail, and then cells were homogenized. Protein concentrations were determined by the method of Bradford [23], using Ultroscpec-2000 UV/VIS Spectrophotometer (Hoefer Pharmacia Biotech Inc, San Francisco, CA, USA). Isolated proteins were run on Western blot or loaded onto dot blot as described previously [19,24]. An amount of 20 μL of each sample was loaded per lane. Lysates from indirect co-cultures were quantified and 15 μg total protein of each sample was loaded per lane. Western blot was normalized to β-actin. To prepare dot blots 200 μL CCM per well was blotted onto a nylon membrane by Minifold-Vacuum-Filtration system SRC-96 (Schleicher&Schuell, Dassel, W. Germany), then subjected to immunoassays. Results were normalized to Ponceau S staining. Primary and secondary antibodies are listed in Table 2.

#### Caseinase and gelatinase zymogram analysis

For caseinase and gelatinase zymogram analysis, 20 μL CCM was used, and normal human serum as control [19,25]. Protease activities were visualized by Coomassie Blue staining (Bio-Rad Laboratories GmbH). Densitometry was carried out on a Kodak Image Station 4000MM Digital Imaging System using Kodak Molecular Imaging Software v. 4.0.3 (Eastman Kodak Company, Rochester, NY, USA).

#### ELISA

CXCL12/SDF-1 and TGF-β1 levels were quantified in mono and co-cultures with the human CXCL12/SDF-1α and TGF-β1 ELISA Kit (R&D Systems, Minneapolis, MN, USA) using 100 μL CCM per sample. As the kit measures active TGF-β1, latent TGF-β complexes were activated before immunodetection as suggested by the manufacturer. ELISA plates were read at 570 nm with Labsystem Multiskan MS 352 (Labsystems, Finland) plate reader.

### Statistical analysis

All experiments were performed in three independent sets, each containing at least three biological replicates. Relative gene expression results were tested for normal distribution by D’Agostino-Pearson omnibus test using GraphPad Prism 4.03 (GraphPad Software, Inc.). Significance of differences between co-culture vs. control values was evaluated by non-parametric tests (Mann–Whitney) and Student’s *t*-tests depending on the distribution of data. Independent experimental sets were then compared for reproducibility. Only reproducible changes with a p < 0.05 level [20] were considered significant. Results of SRB measurements were evaluated by χ^2^ test.

## Results

### Changes in ECM structure of cervical cancer

**Figure 1:**
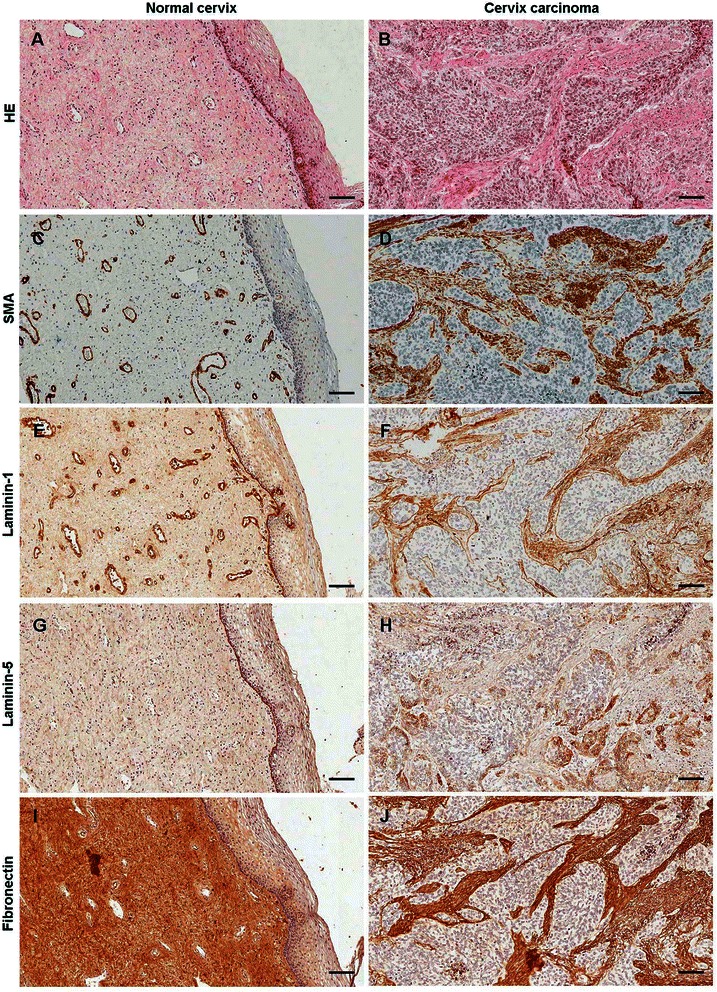
Immunohistochemistry of ECM proteins in the normal cervix and in cervical cancer: H&E staining (**A**, **B**). Smooth muscle actin reaction is detectable in the smooth muscle layer of small blood vessels in the normal subepithelial stoma (**C**), or the activated myofibroblasts in the stroma of cervical cancer (**D**). In the normal tissue laminin-1 is confined to the basement membrane of epithelial layer and small blood vessels (**E**), whereas in the tumor tissue multiple layers are formed around the tumor nests, filling up the stromal regions (**F**). Laminin-5 is present exclusively in the basement membrane of normal epithelium as a single layer (**G**), whereas except for some abortive basement membrane-like formations, the immunopositivity resides in the cytoplasm of tumor cells (**H**). Fibronectin appears as homogenous staining in the connective tissue of normal cervix (**I**), the reaction is distributed in the tumorous stroma in a similar manner (**J**). Scale bars 100 μm.

**Figure 2:**
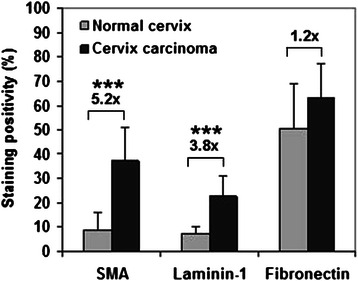
Densitometric evaluation of SMA, laminin and fibronectin of 27 normal and 29 tumorous specimens of tissue microarray. In the tumors the average intensity of SMA immunostaining was 5.2-fold higher, laminin-1 protein expression showed a 3.8-fold increase and fibronectin reaction was similar, showing a non-significant 1.2-fold increase in cervical cancer tissues (black column) as compared with normal (gray column) tissues. The signal positivity is shown as a proportion of the total measured area. Stars indicate significance: ***p < 0.001.

Figure 1A and B shows the characteristic HE staining of normal (A) and tumorous (B) cervix in tissue microarray. SMA positivity was detected in the smooth muscle layer of blood vessel walls in normal subepithelial stroma (Figure 1C), whereas mainly stromal myofibroblasts displayed positivity in tumors (Figure 1D). The intensity of SMA immunostaining was 5.2-fold higher (Mann Whitney test: p < 0.0001) in cervical cancer than in normal tissues (Figure 2). Laminin-1 could be detected in the basement membranes underlying epithelial cells and in the blood vessels in the normal stroma (Figure 1E). In addition to being localized to the same compartments, laminin-1 was deposited in the tumorous interstitial stroma. Incomplete laminin-1 positive basement membrane was seen around the leading edges of tumorous nests (Figure 1F). Compared to normal tissue, laminin-1 protein expression showed a 3.8-fold increase (Mann Whitney test: p < 0.0001) in the tumorous stroma (Figure 2). Laminin-5 was detected in the basement membrane of normal tissues (Figure 1G), while being present in the tumorous area only at the interface of tumor nests and stroma. At the periphery of tumor nests, the cytoplasm of cancer cells was loaded with laminin-5 (Figure 1H). Due to the lack of extensive stromal immunostaining laminin-5 was not evaluated by the software used for other proteins. Fibronectin was evenly and abundantly distributed both in the normal (Figure 1I), as well as in the tumorous stroma with a minor 1.2-fold increase in the cancerous environment (Figures 1J and 2).

### Characteristics of normal and tumorous fibroblasts in tissue cultures

#### Proliferation

**Figure 3:**
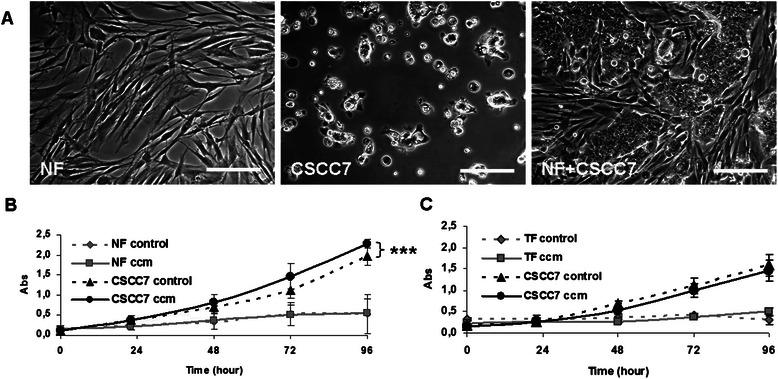
Effect of fibroblasts on the proliferation of CSCC7 cells. CSCC7 cells grew much faster in the presence of normal fibroblasts than alone 48 hours after seeding (**A**). Culture media of normal fibroblasts enhanced the proliferation of CSCC7 tumor cells, indicating the presence of a yet unidentified growth stimulatory compound produced by normal fibroblasts (**B**), whereas the culture media of tumor associated fibroblasts exerted no effect (**C**). Factors produced by the tumor cells did not affect the proliferation of fibroblasts (**B**, **C**). CSCC7 control cells on chart **B** and **C** are taken from two independent experiments. Stars indicate significance: ***p < 0.0001. Scale bars correspond to 100 μm.

The proliferation rate of cells was measured under mono- and co-culturing conditions. When plating them together, fibroblasts surrounded the nests of tumor cells forming a structure that resembled the architecture seen in the tumor tissue. Fibroblasts grew in size and formed spaces between CSCC7 cells (Figure 3A). NF cells exhibited higher proliferation rates than TF fibroblasts. The proliferation of neither normal nor tumorous fibroblasts was stimulated by the conditioned media of cancer cells (Figure 3B-C).

#### Secreted matrix protein profiles

**Figure 4:**
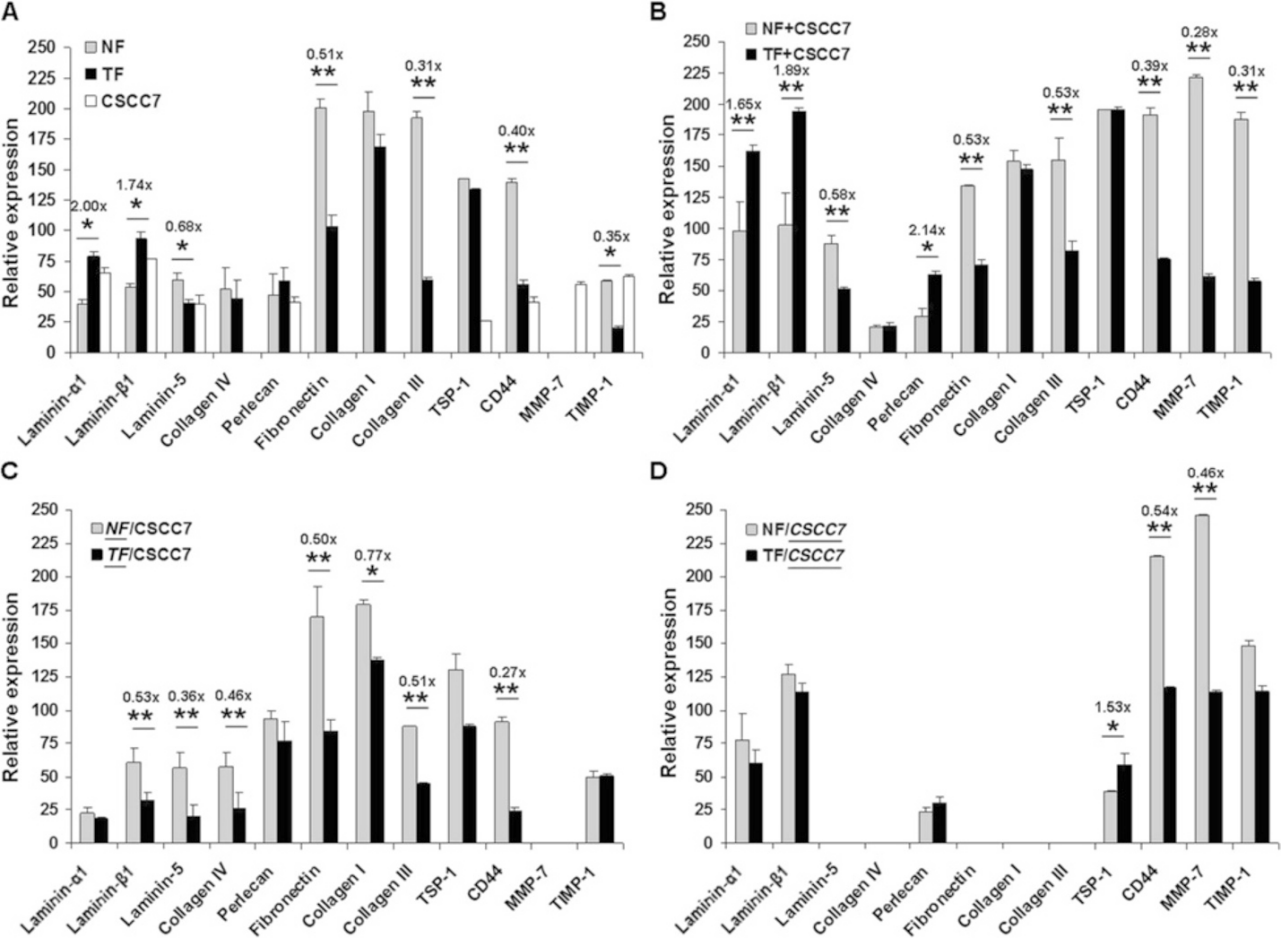
Secreted matrix protein profile of normal and tumor associated fibroblasts growing alone or in co-culture with CSCC7 cells. Dot blots were prepared from the conditioned medium of NF, TF and CSCC7 in monoculture (**A**), from direct co-culture of NF + CSCC7 and TF + CSCC7 (**B**), from the indirect co-culture fibroblast side of *TF*/CSCC7 and *NF*/CSCC7 (**C**), and the indirect co-culture cancer cell side of TF/*CSCC7* and NF/*CSCC7* (**D**). Blots indicate reaction with antibodies against the proteins. The difference in secretory activity between normal and tumorous fibroblasts was assessed. Stars indicate significance: *p < 0.05, **p < 0.001.

Quantitative changes in 11 matrix and secreted proteins were determined by dot blot from the culture media of cells growing alone and in direct or in indirect co-cultures (Figure 4). As a general phenomenon, TFs produced more laminin-α1, −β1 and perlecan and less laminin-5, fibronectin, type III collagen and TIMP-1 than NFs (Figure 4A).

##### Direct contact between fibroblast and CSCC7 cells

The presence of cancer cells resulted in about a twofold increase in laminin-α1 and -β1 both in normal and tumorous fibroblast preparations (Figure 4B versus Figure 4A). The amount of the two laminin chains was significantly higher in TF + CSCC7 as compared with NF + CSCC7 co-culture, and this was true for perlecan as well (Mann Whitney test: p < 0.001). On the contrary, type IV collagen was low in both co-cultures. Interestingly, TSP-1 production of fibroblasts was equal in both sets of samples. In comparison with the medium of NF alone (Figure 4A), the amount of collagens and fibronectin decreased in NF + CSCC7 co-culture, although still being higher than as measured in TF + CSCC7 co-culture (Figure 4B).

##### Indirect contact between fibroblast and CSCC7 cells

In the event NFs and TFs had no physical contact with tumor cells but their media could communicate, no stimulatory effect was observed regarding the laminin chains. Furthermore, TFs secreted less laminin chains than NFs. The levels of fibronectin and fibrillar collagens did not differ significantly from those of control fibroblasts (Figure 4A,C). This observation clearly shows that fibroblasts do not produce MMP7.

##### CXCL12/SDF-1 and TGF-β1 secretion

Concentrations of CXCL12/SDF-1 and TGF-β1 were measured from culture supernatants by means of ELISA. No CXCL12/SDF-1 could be detected in any of our experimental systems. Surprisingly, fibroblasts did not secrete TGF-β1.

#### Activity of matrix remodeling enzymes

**Figure 5:**
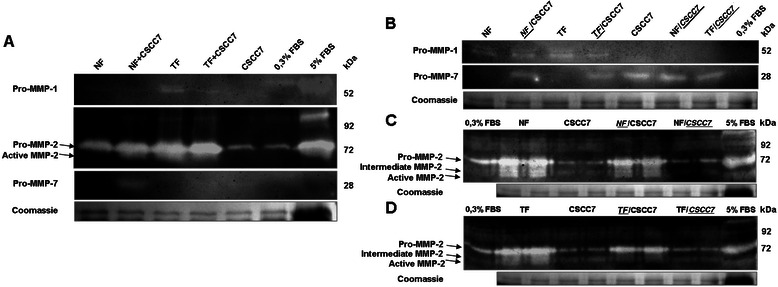
Casein and gelatin zymograms from cell culture supernatants of direct and indirect co-cultures. Casein zymograms show that pro-MMP-1 was produced only by fibroblasts (**A**, **B**) and MMP-7 was produced exclusively by CSCC7 cells (**A**, **B**) in both systems. According to gelatin zymograms MMP-2 was present both in its active and inactive forms (**A**, **C**, **D**) and MMP-9 enzyme activity was not detected in any created setup (**A**, **C**, **D**). In direct co-cultures, the presence of CSCC7 cells activated MMP-2 (**A**). However, in indirect co-cultures the presence of CSCC7 cells reduced the amount of both active and inactive MMP-2, both in case of NFs and TFs (**C**, **D**). Indirect co-culture clearly demonstrates that MMP2 is produced by the fibroblasts and the activity detected in the medium of CSCC7 cells is not higher than that of 0.3% serum, used for the cultivation of tumor cells.

Positive MMP-1, MMP-2 and MMP-7 enzyme activities were found by casein and gelatin zymography of cell culture supernatants of direct (Figure 5A) and indirect (Figure 5B-D) co-cultures. Only fibroblasts exerted pro-MMP-1 activity, which was higher in TFs than in NFs. The pro-MMP-1 activity was reduced in the presence of CSCC7 cells (Figure 5A-B). MMP-2 was present both in its active and inactive forms (Figure 5A and 5C-D). CSCC7 cells in direct co-culture enhanced and activated MMP-2 in the medium of both types of fibroblasts (Figure 5A). However, in indirect co-culture CSCC7 cells did not affect the activity of the enzyme (Figure 5C-D).

#### Expression of ECM binding membrane proteins and receptors

**Figure 6:**
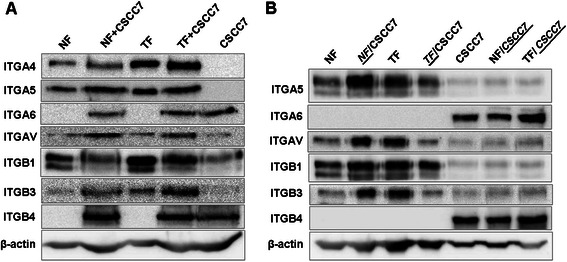
Integrin Western blot results in direct and indirect co-cultures. In case of fibroblasts, mainly thrombospondin (α4β1 αvβ3), fibronectin (α5β1, αvβ1, αvβ3), tenascin (αvβ3), vitronectin (αvβ3, αvβ5) and VCAM-1 (α4β1) binding integrin pairs are visible, while tumor cells only show weak binding, with the expression of mostly laminin-binding integrin pairs (α3β1, α6β1, α6β4). Panel **A** shows Western blot results of integrins in direct co-culture, and panel **B** in indirect co-cultures.

Integrin expressions in direct (Figure 6A) and indirect (Figure 6B) co-cultures were examined by immunoblot. Signals were normalized to those of β-actin. Integrins α4 and α5 were expressed exclusively by fibroblasts, whereas integrins αv, β1 and β3 by both cell types (Figure 6).

On the whole, in case of TFs integrin expression was higher than in case of NFs. With the exception of integrin β1, direct contact with tumor cells resulted higher levels of integrin expression in both NFs and TFs. Expressions of αv and β3 integrins were upregulated in co-cultures indicating the importance of this integrin pair in the cooperation between fibroblasts and tumor cells (Figure 6A). Indirect contact was capable of stimulating integrin expression in case of NFs, whereas TFs became unresponsive (Figure 6B).

**Figure 7:**
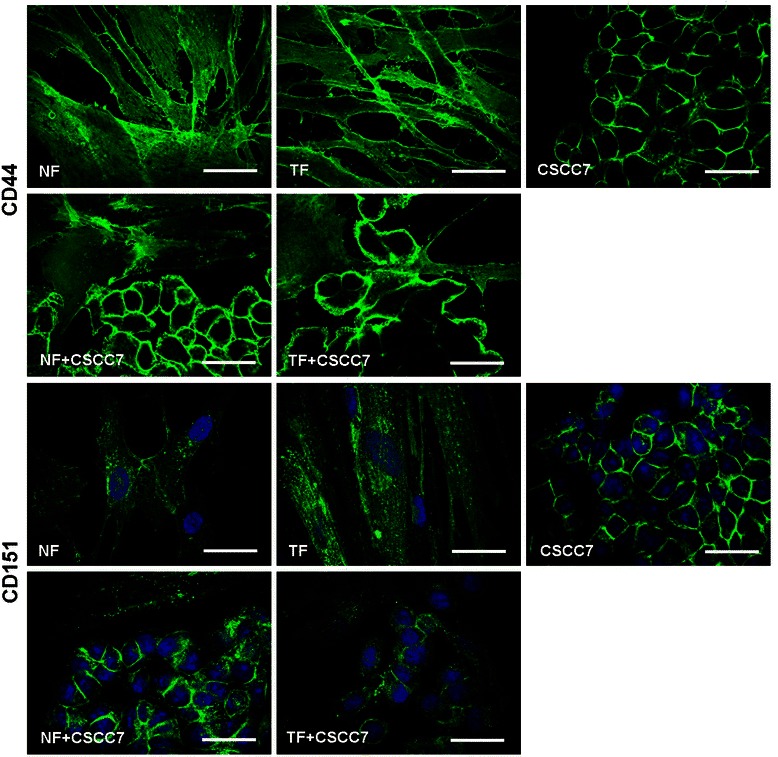
CD44 and CD151 immunocytochemistry. NF, TF and CSCC7 cells express comparable amounts of CD44 on their cell surface. CD151 was similarly positive on fibroblasts and tumor cells. Strong staining was detected between tightly packaged CSCC7 cells in tumor nests, which faded on the surface of scattered tumor cells separated by TF cells in direct co-cultures.

Despite Figure 7 showing that both NFs and TFs expressed comparable amounts of CD44 on their cell surface, dot blots demonstrated the shedding activity of this cell surface glycoprotein in case of both fibroblasts. This activity was found to be much higher in case of NFs than in case of TFs, either alone or growing in direct and indirect co-culture (Figure 4).

The surface protein CD151 was equally positive on fibroblasts and tumor cells as indicated by immunostaining (Figure 7).

### Response of tumor cells to the presence of fibroblasts

#### Proliferation

Figure 3A illustrates that 48 h after seeding CSCC7 cells growing in direct co-culture contained a significantly larger amount of tumor cells than those growing alone and formed nests that were surrounded by normal fibroblasts. Compared to fibroblasts, their proliferation rate was higher and showed further increase when exposed to CCM of NF cells for 96 h (Student’s *t*-test: p < 0.0001) (Figure 3B). The CCM of TF cells did not exert the same effect (Figure 3C).

#### Secreted matrix protein profiles

According to our results, CSCC7 cells synthesized laminin-α1, −β1, −5, perlecan, TSP-1, CD44, MMP-7 and TIMP-1 (Figure 4A). When growing in direct co-culture with NFs, these cells strongly upregulated MMP-7 production (Figure 4B). Even in spatial separation, the co-culture with fibroblasts seemed to induce the synthesis of laminin-1 by cancer cells (Figure 4D). The lack of laminin-5 in the medium was in agreement with the immunostaining shown in Figure 1H displaying the protein inside the cytoplasm of tumor cells.

**Figure 8:**
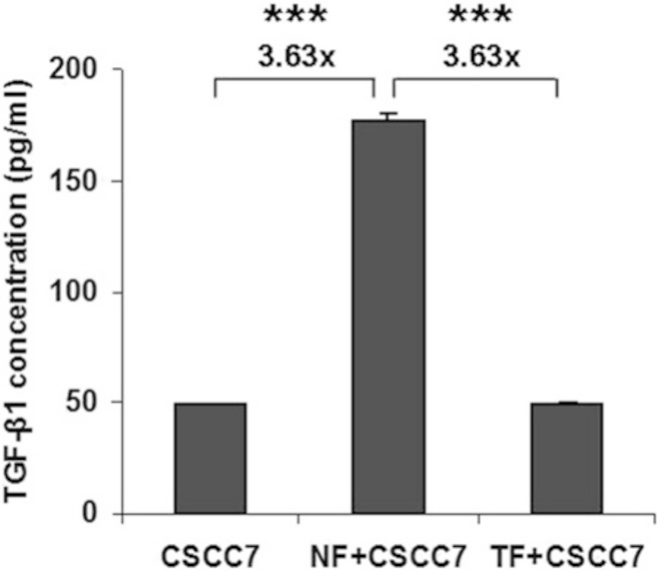
TGF-β1 ELISA results. ELISA suggests that only CSCC7 cells secrete TGF-β1. Direct co-cultivation of tumor cells with NF cells stimulates synthesis of the growth factor. TF cells failed to force TGF-β1 production of CSCC7 cells.

Under all conditions, including monoculture, direct- and indirect co-culture, CCMs of CSCC7 cells contained active TGF-β1 (~50 pg/mL). Physical contact with NFs, but not with TFs significantly increased the level of active TGF-β1 (3.63-fold, Mann Whitney test: p < 0.0001), in case of the NF + CSCC7 medium (Figure 8).

#### Activity of matrix remodeling enzymes

The levels of protein expression and activity of MMP-7 were quantified (Figure 5). According to both measures, MMP-7 was produced only by CSCC7 cells. In direct co-culture, pro-MMP-7 activity and protein production were enhanced only in the presence of NFs (Figure 5A). Neither MMP2 nor MMP-9 activity was detected in tumor cells (Figure 5A and 5C-D).

#### Expression of ECM binding membrane proteins and receptors

Integrins α6 and β4 were found exclusively on tumor cells. Integrins αv, β1 and β3 were present both on fibroblasts and tumor cells (Figure 6). The presence of TF cells stimulated the expressions of integrin α6 and β4 as well as integrin αv, β1 and β3 (Figure 6B).

In addition to integrins, the standard form of CD44, another ECM binding receptor, was able to form a complex with MMP-7 as detected by immunocytochemistry. As also shown by immunohistochemistry (Figure 7), tumor cells expressed ample amounts of CD44 on their surface. CD44 was found to be shed actively into the medium as a result of co-culture with NFs (Figure 4D).

The immunocytochemical staining of the integrin stabilizing cell surface protein CD151 is shown in Figure 7. Strong staining was detected between tightly packed CSCC7 cells in tumor nests, which faded on the surface of scattered tumor cells separated by TF cells in direct co-culture.

#### Invasion and migration

**Figure 9:**
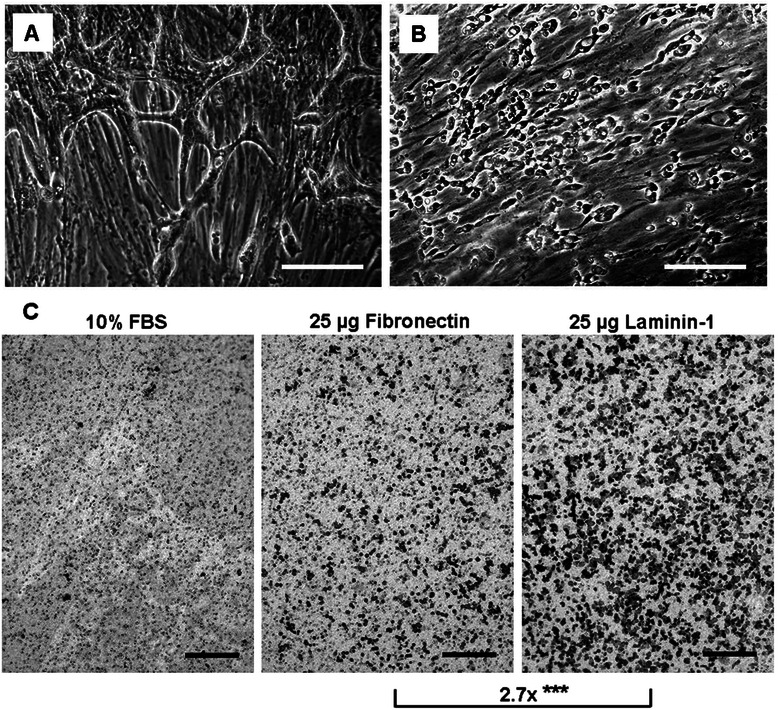
Invasion and migration of tumor cells. Primary (**A**) and immortalized (**B**) tumor cells can be seen spread on the fibroblast layer. Tumor cells failed to form groups, preferably dwelling as single cells. Scale bars: 100 μm. CSCC7 cell migration was moderate for fibronectin and more intense for laminin-1 in a Boyden chamber after 24 hours (**C**). Scale bars: 200 μm, stars indicate significance: ***p < 0.001.

In addition to fibroblasts, tumor cells were found to adhere and start to proliferate in the primary cell cultures established from tissue explants. Since a prerequisite for tumor cell survival and growth is the prior lawn formation of fibroblasts, the tumor cells in these cultures were easily distinguishable from other cells owing to the fact that immune cells do not survive cultivation, so they could be excluded. Benign cells, like fibroblasts do not grow on top of each other, this is a characteristic feature of malignant cells. The morphological finding of cells spreading on the fibroblast lawn clearly corresponded to cancer cells by reason of their variable sizes and irregular features, large nuclei, prominent nucleoli, decreased cytoplasm/nucleus ratio all of which are the criteria of malignancy. In addition, vimentin and pan-cytokeratin staining served to differentiate between cell types, corroborating our morphological observations, which imply that the presence of tumor-associated fibroblasts is a fundamental requirement for the growth of tumor cells. Instead of node formation, single tumor cells were seen to spread and migrate on top of the fibroblast layer (Figure 9A). A similar phenomenon was observed when CSCC7 cells were seeded on top of a confluent sheet of tumorous fibroblasts, inhibited by mitomycin C to grow. In these cases tumor cells failed to form groups, preferring rather to dwell and migrate in the form of single cells (Figure 9B).

To identify the most potent chemoattractant for tumor cell migration, mitomycin C treated CSCC7 cells were allowed to migrate toward various chemoattractants in a Boyden chamber for 24 h. The medium, conditioned by co-cultured cells, attracted tumor cell migration very inefficiently. Laminin-1 proved to be the strongest chemoattractant, as the number of tumor cells migrating towards laminin-1 increased by 2.7-fold as compared with fibronectin (Mann Whitney test: p < 0.0001) (Figure 9C).

## Discussion

The tumor microenvironment plays a pivotal role in the behavior of cancer [26]. To identify the characteristic changes of ECM as a result of tumor-matrix interactions we established a tissue microarray from cervical cancer specimens. One of the most striking changes noticeable after cancerous transformation was the upregulation and redistribution of laminin-1, depositing not only into basement membranes, but also into the fibrillar connective tissue. In line with this observation, SMA revealed strong stromal positivity, indicating the presence of activated myofibroblasts that are the major producers of ECM in the stroma [27]. In support of earlier findings, laminin-5 resided in the cytoplasm of cancer cells [28].

To obtain more information about the functions shared between fibroblasts and tumor cells, we established fibroblast cultures from tumor free cervix (normal fibroblasts) and tumorous parts of surgically removed cancerous uterine cervix (considered as tumor-associated fibroblasts). An established HPV16 positive cervical cancer cell line, CSCC7, served as the tumorous variance [18].

When grown alone, comparison of NF and TF cells showed NFs to predominantly synthesize components of the interstitial matrix, such as fibronectin and type I and III collagens. When placed into direct co-culture, only NFs were able to stimulate cancer cells to produce TGF-β1 and MMP-7, factors needed for proliferation and local invasion [5]. The conditioned medium of NFs but not TFs was capable of stimulating the proliferation of tumor cells.

Compared to NFs, TFs were reprogrammed to produce increased amounts of laminin-α1 and -β1. These are components of laminin-111 known to be implicated in cancer progression, which contain peptide sequences active in proliferation, angiogenesis and metastasis [29]. Furthermore, TF synthesized a smaller amount of type IV collagen, a protein which is prerequisite for the formation of natural basement membranes. The secretion of fibrillar matrix components was found to be decreased.

Direct co-culture of tumor cells with TFs resulted in the further increase of laminin-1 and perlecan and a decrease in type IV collagen secretion. Thus, it appears that cervical cancer cells are in need of a laminin-rich fibrillar matrix for their invasion, creating an imbalance between components of basement membrane molecules. The fact that laminin-α1 and -β1 production stayed low when TFs were growing in indirect co-culture indicates that direct contact with tumor cells is needed for the enhancement of laminin synthesis. On the contrary, tumor cells were found to be capable of maintaining high laminin synthesis under such conditions, compensating for the low production of fibroblasts.

The importance of laminin-111 in the pathology of cervical cancer was underlined by the facts that laminin proved to be the most efficient chemoattractant in the Boyden chamber migration assay and that it was also produced by CSCC7 cells. These cells predominantly expressed α6β4 and α6β1 integrins, two laminin binding receptors [30]. This observation implied that laminin-integrin interaction took place both in paracrine and autocrine fashion. In addition to laminin-111, tumor cells growing alone secreted laminin-5, perlecan, MMP-7, TIMP-1 and CD44. Although the current assay was limited to immuncytochemistry, strong expression of CD151 was found, a protein known to be involved in the stimulation of laminin receptor-associated invasion, and activation of MMPs was also noted, with both observations manifest on the surface of TF and CSCC7 [31-33].

These results denote that the two types of fibroblasts exhibit different actions as regards the proliferation and migration of cancer cells. NFs stimulated cell proliferation whereas TFs promoted migration. Moreover, it was found that the presence of secreted fibroblasts and matrix proteins was fundamental for the survival of tumor cells in primary tissue culture. A similar observation was published by *Maffini et al.* who showed that only tumor cells injected together with activated fibroblasts are able to colonize in case of mice [34].

It would seem that the differences between NFs and TFs represent the stages of fibroblast transformation from defensive to permissive cells. It can not decidedly be said that the so-called “normal fibroblasts” would indeed represent a defensive cell population. Nevertheless, the fact that they promote cell proliferation suggests that they are on the road to fall into line with supportive mechanisms of cancer. Furthermore, synthesized αv integrin is able to activate latent TGF-β1 alone or by targeting it toward MMPs [35].

Since MMPs facilitate tumor cell invasion and metastasis, in the current study we assessed MMP activities of fibroblasts and tumor cells. Zymography revealed that three MMPs were involved in cervical cancer progression. Fibroblasts, especially TFs, secreted pro-MMP-1, responsible for the digestion of type I and type III collagens, and pro- and active MMP-2 which is implicated in the degradation of the basement membrane. In support of earlier studies, CSCC7 cells produced pro-MMP-7 [36,37]. MMP-7, in turn, was found to regulate the angiogenic activity of fibroblasts [38]. Among MMPs. only MMP-2 was present in active form. Direct contact between fibroblasts and tumor cells upregulated the secretion and activation of MMP-2, but only NFs increased TIMP-1 production.

Worldwide, HPV16 and HPV18 contribute to over 70% of all cervical cancer cases [39], therefore the HPV16 positive CSCC7 tumor cell line was selected for the current study. A number of reports propose that CD151 and integrin α6 have key roles in HPV16 infection of epithelial cells [40-42]. In the current study, although using immunocytochemistry alone, we found strong expression of CD151, both in fibroblasts and cancer cells. This protein is involved in the laminin-binding of various integrin pairs [43] and in the stimulation of laminin receptor-associated invasion, by stabilizing integrins α3β1 and α6β4, which is a requirement for motility of invasive tumor cells [44,45]. In addition, CD151 is capable of binding and activating MMPs, including pro-MMP-7 onto the cell membrane, facilitating ECM degradation [31-33,46].

We found another molecule, TSP-1, to be critical in the pathology of cervical cancers. TSP-1 is a factor participating in cell adhesion-antiadhesion [47] and is implicated in tumor progression and angiogenesis. It upregulates integrin α6 in keratinocytes and breast cancer cells resulting in an increased level of cell adhesion and tumor cell invasion [48]. Of interest is the rather similar behavior of this molecule in the two types of fibroblasts, which may indicate its perpetual importance in tumor-stromal interaction. We detected α4 and β1 integrins on the surface of both types of fibroblasts, an integrin pair known to bind thrombospondin-1 [49]. In this context, this glycoprotein is capable of exerting its actions on the surface of fibroblasts. Earlier reports support the importance of thrombospondin-1 in the activation of latent TGF-β1 as well as in the stimulation of EGFR by its EGF-like domains [50].

The efficient adhesion and directional movements of tumor cells migrating on the surface of a fibroblast layer can be explained by the ordered structure of ECM facilitated by the expression of syndecan-1 on the surface of fibroblasts [51]. In fact, the presence of syndecan-1 is a typical feature of fibroblasts isolated from cervical cancers (unpublished result of the authors). Furthermore, migratory phenotype is attributed to the mesenchymal expression of CD44, demonstrated both on NF and TF cell surfaces [52]. Accordingly, we were able to demonstrate that physical contact between fibroblasts and CSCC7 cells initiates tumor cells to start migrating. Soluble CD44, probably shedding due to increased MMP-7 protease activity, promotes the effect further by inhibiting adhesion of tumor cells [53].

## Conclusions

In summary, we were able to demonstrate the differences between NFs and TFs in selectively expressing factors required for proliferation or invasion of cervical cancer cells. The most significant effects of normal fibroblasts when attracted to tumor cells were the production of protein molecules such as TSP-1 promoting anti-adhesion, tumor cell proliferation and, together with integrin αvβ3, the activation of TGF-β1 [54]. Thus, MMP-2 was secreted and MMP synthesis was facilitated, resulting in degradation of the basement membrane. At the same time, TFs facilitated laminin-1 synthesis, promoting the migration of tumor cells that expressed laminin receptors. MMP7 produced by tumor cells cleaved to the extracellular domain of CD44, which is a participant in the creation of an anti-adhesive milieu for tumor migration.

## Abbreviations

SMA: Smooth muscle actin
CSCC7: Cervical squamous cell carcinoma 7
MMPs: Matrix metallo-proteases
TGF-β1: Transforming growth factor beta 1
CAFs: Cancer associated fibroblasts
ECM: Extracellular matrix
HA: Hyaluronan
SCC: Squamous cell carcinoma
EMT: Epithelial mesenchymal transition
HPV: Human papilloma virus
TMA: Tissue microarray
HE: Hematoxylin- and eosin-stained
NF: Normal fibroblast
TF: Fibroblasts derived from a tumorous area
CCMs: Conditioned culture media
CLs: Cell layers
SRB: Sulforhodamine B
SDF-1: Stromal cell-derived factor 1 (CXCL12)
TIMPs: Metallopeptidase inhibitors
CD151: Tetraspanin
TSP-1: Thrombospondin 1
EGFR: Epidermal growth factor receptor
